# Understanding multi‐stakeholder needs, preferences and expectations to define effective practices and processes of patient engagement in medicine development: A mixed‐methods study

**DOI:** 10.1111/hex.13207

**Published:** 2021-02-17

**Authors:** Stuart D Faulkner, Suzanne Sayuri Ii, Chi Pakarinen, Fabian Somers, Maria Jose Vicente Edo, Lucia Prieto Remon, Ana Diaz Ponce, Dianne Gove, Elisa Ferrer, Begonya Nafria, Neil Bertelsen, Mathieu Boudes, Nicholas Brooke, Alexandra Moutet, Nick Fahy

**Affiliations:** ^1^ Radcliffe Primary Care Building Radcliffe Observatory Quarter Oxford UK; ^2^ The Synergist Brussels Belgium; ^3^ UCB Brussels Belgium; ^4^ Aragon Health Sciences Insitute Instituto Aragonés de Ciencias de la Salud (IACS) Zaragoza Spain; ^5^ Alzheimer Europe Luxembourg; ^6^ EURORDIS‐Rare Diseases Europe Paris France; ^7^ Patient Engagement in Research Institut de Recerca Sant Joan de Déu‐ Hospital Sant Joan de Déu Barcelona Spain; ^8^ Patient & Citizen Involvement in HTA Group HTAi; ^9^ European Patients’ Forum (EPF) Etterbeek Belgium

**Keywords:** expectations patient engagement, health technology assessment bodies, medicines development, potentially vulnerable populations, stakeholder expectations patient engagement

## Abstract

**Background:**

The holistic evolution of patient engagement in medicines development requires a more detailed understanding of the needs of all involved stakeholders, and one that better accounts for the specific needs of some potentially vulnerable patient populations and key stages in medicines development.

**Objective:**

The purpose of this convergent mixed‐methods study was to better understand the needs of different stakeholders concerning patient engagement at three key stages in medicines development: research priority setting, clinical trial design and early dialogues with Health Technology Assessment bodies and regulators.

**Design:**

This study brought together findings from three sources: i) an online questionnaire, ii) face‐to‐face consultations with two potentially vulnerable patient populations, a workshop with Health Technology Assessment bodies, and iii) three‐step modified Delphi methodology.

**Results:**

Overall stakeholders still need additional varied support mechanisms to undertake, sustain or measure value of patient engagement. Health Technology Assessment bodies need better rationale for patient engagement in early dialogue and tools to support its implementation. Improved awareness and understanding of the need and value that involving patients, who are often considered as potentially vulnerable, can bring is needed, as is better accommodation of their specific needs. Similarly, weighted Delphi categories were as follows: aims and objectives, and sustainability. Several additional themes were common across the three key stages in medicines development.

**Conclusion:**

This broad‐reaching study provides the blocks needed to build a framework for patient engagement in medicines development.

**Patient or Public Contribution:**

Patients were involved in review and interpretation of data.

## 1 INTRODUCTION

There is increasing consensus among stakeholders that patient engagement (PE) in medicines development is critical to fostering patient access to better designed innovative therapeutic solutions and delivering more effective health outcomes for patients.^1^, ^2^, ^3^, ^4^, ^5^, ^6^ Key involved stakeholders include the patient community (patients, patient advocates and patient organizations), the pharmaceutical industry, regulators, health technology assessment (HTA) bodies, payers, health‐care professionals (HCP), academia and to a certain extent policymakers and research funders. Although there are different perspectives on what PE means, here we define PE as, ‘the effective and active collaboration of patients, patient advocates, patient representatives and/or carers in the processes and decisions within the medicines lifecycle, along with all other relevant stakeholders when appropriate’.^7^ While there are many initiatives to involve patients across that continuum, inconsistency and fragmentation remain the norm.^1^, ^8^, ^9^, ^10^ This is especially so for the meaningful engagement of potentially vulnerable patient populations who often have additional or specific needs (such as people with dementia and young people).^11^, ^12^ Patient engagement also needs to be understood better at key upstream stages of medicines development that are comparatively poorly serviced by current efforts such as research priority setting, clinical trial design and early dialogues with health technology assessment (HTA) bodies and regulators. Achieving systematic and meaningful PE are challenging and must meet the needs, expectations and context^13^, ^14^ of the different stakeholders involved.^3^, ^8^, ^15^, ^16^ A better understanding of multi‐stakeholder needs, preferences and expectations for patient engagement would provide a more solid foundation for empowering actions such as the creation of tools and frameworks that can holistically enhance effective, meaningful and sustainable PE.

### 1.1 Aims and objectives

We aimed to generate criteria for effective PE in medicines development, focused on three key stages where systematic PE is generally less mature compared to other stages and taking into consideration the specific needs of, and support that should be in place for, potentially vulnerable patient populations, and which are not always appropriately addressed in current PE approaches. These key stages and patient populations were as follows: (i) research priority setting (RPS) – providing opinion, providing evidence and/or being part of a group that decides what is important to research, (ii) clinical trial design (CTD) – designing protocols, discussing patient burden, discussing patient‐related outcomes, iii) early dialogues with regulators and HTA bodies (ED) – early discussions between industry, HTA bodies and regulators (and in some contexts with payers) regarding developmental plans for a medicinal product and to ensure they meet the requirements and iv) potentially vulnerable patients, in the context of this project, include (but are not limited to) people with dementia and their carers, and young people.

## 2 DESIGN AND METHODS

**Figure 1:**
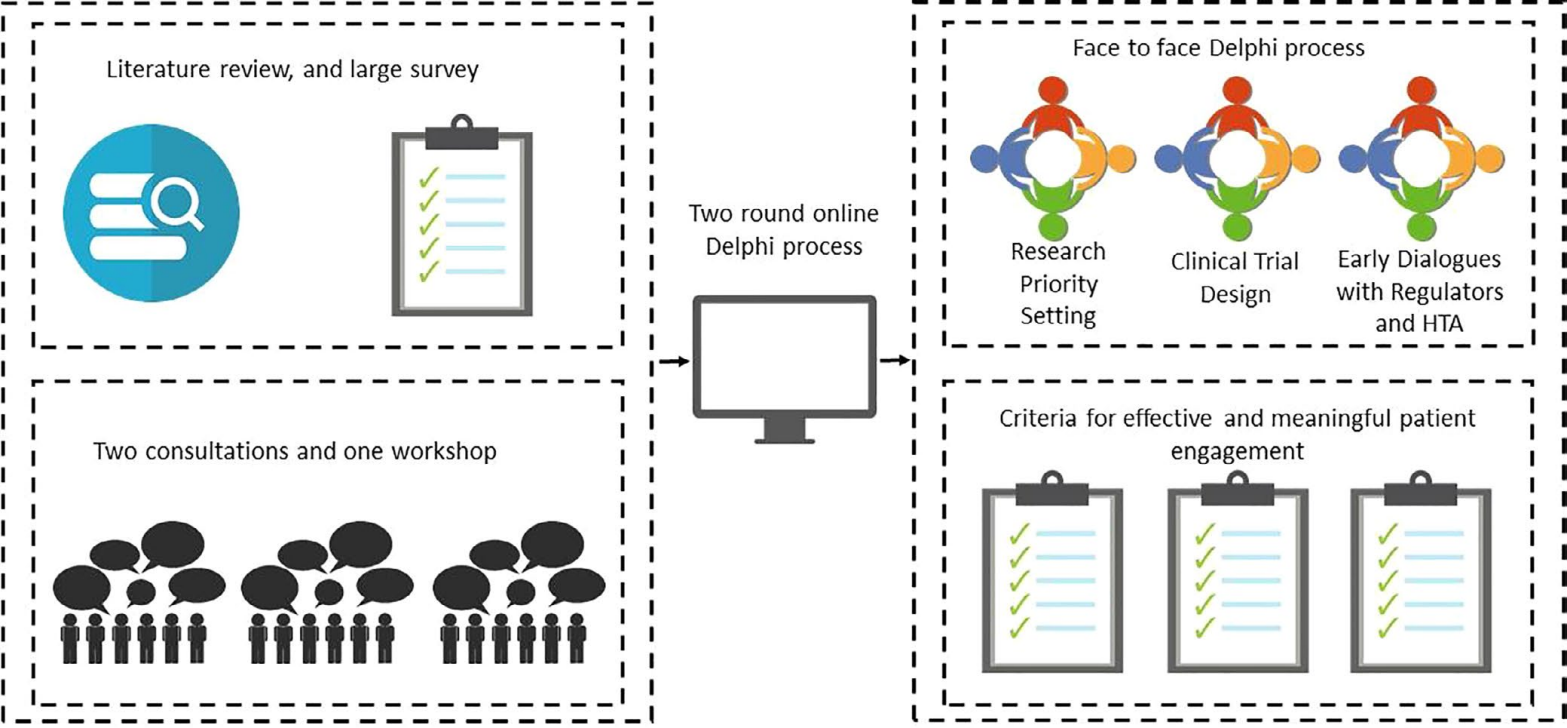
Infographic of three‐stage convergent methodology. The method contained three components – each informing the next: i) an online questionnaire to capture needs, expectations and preferences, ii) face‐to‐face consultations with two groups of potentially vulnerable patient populations, and a separate workshop with representatives from HTA bodies, and iii) three‐step Delphi exercise to identify minimal criteria for effective and meaningful PE

This work was conducted within the context of the PARADIGM project, funded by the Innovative Medicines Initiative (IMI), that developed ways to ensure that patients are always meaningfully involved in the development of medicines.^7^ This study took a convergent mixed‐methods approach combining quantitative methods and a qualitative approach, using consultations set within the framework of patient and public involvement (PPI). The learning and key emerging themes from each stage were incorporated into each following stage. This brought together three components: i) an online questionnaire to capture overall identification of needs, expectations and preferences of effective PE from all involved stakeholders, ii) separate face‐to‐face consultations with two specific groups of potentially vulnerable patient populations, and a separate workshop with representatives from HTA bodies – to gather greater insight on the needs and preferences of these groups beyond the results of a survey, and (iii) modified Delphi exercise to identify and prioritize the minimum agreed criteria for effective and meaningful PE at the three key stages of medicines development (Figure 1).

PARADIGM consortium partners AE and FSJD sourced patients involved in the two consultations from pre‐existing working groups within each respective umbrella organization. Each was led by their own staff and followed its own standard institutional processes for organizing PPI activities.^17^, ^18^ Delphi panel experts were identified from the PARADIGM consortium networks, and standard institutional processes were followed for undertaking the Delphi exercise.

The separate workshop with HTA bodies was overseen by the HTA international (HTAi) Patient & Citizen Involvement Group (PCIG) Steering Committee. For the network of HTA agencies and the European Network for HTA (EUnetHTA) Early Dialogues Working Party (EDWP), the French HTA agency, Haute Autorité de Santé (HAS), as the co‐lead of this group, sought approval from the HAS board for involvement in this project (see *Bertelsen N, et al 2020, manuscript under submission to Health Expectations*).

### 2.1 Stage 1: Survey

This survey aimed to identify current needs and expectations for PE across medicines development. The survey was constructed in two phases. Firstly, issues identified from existing literature on PE in medicines development^8^, ^15^, ^16^, ^19^, ^20^ were prioritized during a face‐to‐face workshop involving a multi‐stakeholder working group. This informed the structure of a survey which was piloted over two weeks using respondents from each respective stakeholder group.

The final survey was made up of 15 general questions that all stakeholder groups completed. Within this general section, two questions were constructed in matrices allowing more than one choice per row, six questions were structured as visual analogue scales (assigning 0‐100 points based on respondents’ impressions on PE) and four questions were multiple‐choice, allowing for more than one option to be chosen. Stakeholder groups: the patient community, industry, regulators, policymakers, HTA bodies and payers, research funders and HCPs (clinical academics) also had additional separate sections comprising matrices and multiple‐choice questions. Within the survey, we initially sought to gain a broad benchmark of PE across the medicines lifecycle, including dialogues with regulators and HTA bodies that involve the licensing of medicines, HTA assessment, and pricing and reimbursement. Hereafter, the workshop with HTA bodies and Delphi panels only explored the definition of early dialogues with regulators and HTA.

The survey was administered in English in an online tool, Survey Gizmo, over a four‐week period in 2018. A snowball technique was utilized to cascade the survey within consortium members’ internal and external networks to reach an estimated sample population of 10,000 (with 95% CI), with a minimum calculated sample size needed of 370.

All key stakeholders in medicines development were targeted: regulators, HTA bodies, industry (pharmaceutical, biotechnology and medical technology companies) health‐care professionals (clinical academics), patients and patient representatives (from disease‐specific or agnostic organizations, and non‐affiliated individual patients), policymakers, research funders and research and academia (research institutes and universities).

Completed survey responses were converted and analysed using a statistical software package (IBM SPSS Statistics, Version 25.0, Amrock, NY: IBM Corp.) and Microsoft Excel (Version 16.20). Findings are described as total responses or percentage of respondents to a given question or theme.

### 2.2 Stage 2: Face‐to‐face consultations and workshops

The specific needs, expectations and preferences of young people, and people with dementia and their carers were explored through separate face‐to‐face consultations, involving respective experienced staff, in order to better understand the specific PE needs of these groups of patients.

In the case of people with dementia, members of Alzheimer Europe's (AE) European Working Group of People with Dementia (EWGPWD)^21^ and their carers participated in a one‐day consultation (total of 11 people with dementia and 10 carers from 10 countries ^22^). Separately, in the case of young people, members of the KIDS Barcelona group – the Young Persons’ Advisory Group (YPAG) of the Sant Joan de Déu Research Foundation (FSJD) participated in a one‐day consultation (total of 14 participants aged 15 to 18 years old).^23^, ^24^ A topic guide, questions and vignettes were used to facilitate discussions. All participants were invited to review the resulting summary report. In the case of the KIDS Barcelona YPAG, a summary of the session was jointly developed and agreed among all attendees.

Given the distinctive and varied nature of HTA bodies and the questions to be explored, a separate scoping meeting preceding a one‐day workshop was held with a variety of HTA bodies to define the topic guide and structure of the main workshop. The later workshop was facilitated by two experienced staff with 11 representatives from HTA bodies that conduct ED with and without patient involvement and some that do not conduct ED at all. HTA bodies were recruited via two existing networks; the HTAi Patient & Citizen Involvement Group (PCIG) network of HTA bodies and the European network for HTA (EUnetHTA) early dialogues working group (EDWP). The workshop explored the rationale for involving patients in ED, challenges with doing so, and prioritized some tools and methods that are needed to better facilitate PE by HTA bodies (see Table 4 for details of HTA representation, and for full methods see *Bertelsen N, et al, 2020, manuscript under submission in Health Expectations*).

### 2.3 Stage 3: Three‐step RAND modified Delphi methodology

The modified Delphi methodology is a recognized method for prioritization of diverse variables through consensus.^25^, ^26^ Delphi questionnaires were developed based on the outputs from stages one and two, combined with a review of existing frameworks for PE.^10^ Experts for each of the Delphi panels were convened using a snowball method. Experts had to hold recognized expertise and experience within their topic panel in relation on PE to guarantee that the group reflected the view of a majority (initial expert group size; RPS = 24, ED = 26, CTD = 31) (Table 1).

**Table 1:** Overall representativeness of the three stage, three panel Delphi experts

Delphi panel	Round 1		Round 2		Round 3		Profiles of Stakeholders in Face‐to‐Face meetings
	Sex	Age (years)	Sex	Age (years)	Sex	Age (years)	
Setting Research Priorities	Women: 13 (65%) Men: 7 (35%)	<25:1 (5%) 35‐44:4 (20%) 45‐54:10 (50%) 55‐64:4 (20%) >65:1 (1%)	Women: 11 (68.7%) Men: 5 (31.2%)	<25:1 (6.25%) 35‐44:2 (12.5%) 45‐54:9 (56.2%) 55‐64:3 (18.7%) >65:1 (6.2%)	Women: 6 (60%) Men: 4 (40%)	<25:1 (10%) 35‐44:2 (60%) 55‐64:1 (10%)	Patients/Patient advocates/Patient representative, Pharmaceutical industry professional, HCP, Research and Academia, Regulator, Research Funder, Professional, Other
Total	20		16		10		
Clinical Trial Design	Women: 22 (75.8%) Men: 7 (24.1%)	<25:1 (3.4%) 25‐34:2 (6.8%) 35‐44:5 (17.2%) 45‐54:18 (62.1%) 55‐64:2 (6.8%) >65:1 (3.4%)	Women: 20 (76.9%) Men: 6 (23.07%)	<25:1 (3.8%) 25‐34:2 (7.6%) 35‐44:5 (19.2%) 45‐54:17 (65.3%) 55‐64:1 (3.8%)	Women: 14 (77.7%) Men: 4 (22.2%)	<25:1 (5.5%) 25‐34:1 (5.5%) 35‐44:4 (22.2%) 45‐54:11 (61.1%) 55‐64:1 (5.5%)	Patients/Patient advocates/Patient representative, Pharmaceutical industry professional, HCP, Research and Academia, HTA body, Research Funder, Other
Total	29		26		18		
Early Dialogues with HTA and regulators	Women: 14 (70%) Men: 6 (30%)	25‐34:1 (5%) 35‐44:4 (20%) 45‐54:10 (50%) 55‐64:5 (25%)	Women: 13 (72.3%) Men: 5 (27.7%)	25‐34:1 (5.5%) 35‐44:3 (16.6%) 45‐54:9 (50%) 55‐64:5 (27.7%)	Women: 9 (75%) Men: 3 (25%)	25‐34:1 (8.3%) 35‐44:3 (25%) 45‐54:4 (33.3%) 55‐64:4 (33.3%)	Patients/Patient advocates/Patient representative, Pharmaceutical industry professional, HTA body, Research and Academia, Regulator, Other
Total	20		18		12		

Note

Rounds one and two were conducted online. Round three was face‐to‐face. Where possible expertise, geographical spread and sex were balance throughout. Research Priority Setting (RPS): Denmark, Spain, Germany, Ireland, Italy, Netherlands, USA, France, Czech Republic and United Kingdom; Clinical Trial Design (CTD):United Kingdom, Germany, Belgium, Spain, Austria, Denmark, Canada, Netherlands, USA and France; Early dialogues with Regulators and Health technology Assessment bodies (ED): United Kingdom, Spain, Italy, Canada, France, Greece, Belgium and Sweden.

Patients (including carers), and Patient advocates and patient organizations. Health‐care professionals (HCP – clinical academics), Health Technology Assessment bodies (HTA), pharmaceutical companies (Pharma), Industry and Biotechnology companies (Biotech), other (include, but not limited to individuals that identify as their primary affiliation of; charity, consultant, independent expert, think tank, industry association, social association, funder, NGO’s, or identified as having multiple relevant affiliations)

Each panel was balanced as far as possible for geographical coverage and sex (see Table 1 and^27^ for additional results). The three Delphi panels were run in parallel and consisted of three rounds of progressive prioritization of criteria and their corresponding categories. Rounds one and two were conducted via an online survey, and round three was a face‐to‐face meeting. During rounds one and two, experts were asked to judge individually how relevant each question was using a Likert scale [from 1 (not relevant at all) to 9 (highly relevant)]. Mean, median, coefficient variation (CV), interquartile range (IQR) and quartile 1 (Q1) were calculated along the vote distribution. Agreement was reached for those questions when Q1 ≥ 7, IQR ≤ 1, CV < 20%; and when the median and the votes fitted within the same bracket (agreement converged around ‘relevant’) by the experts. Questions were dropped when Q1 ≤ 6, IQR ≥ 2, CV > 20% and there was agreement when the median and votes fitted within the ‘irrelevant’ or ‘not clearly relevant’ bracket. The rest of the questions that did not reach any agreement were kept for reassessment for the second round where participants were given round one results and asked to individually re‐score and order the categories – this included questions where previously no agreement was reached. Round three aimed to i) reach consensus among the items on which there was disagreement; ii) merge and rephrase criteria and categories*;* iii) weight categories within criteria; and iv) weight individual criteria.

## 3 RESULTS

Here we present the findings of each of the three stages and the overall conclusions. Additional results are available athttps://imi‐paradigm.eu/our‐work/.^28^

### 3.1 Survey general characteristics

**Figure 2:**
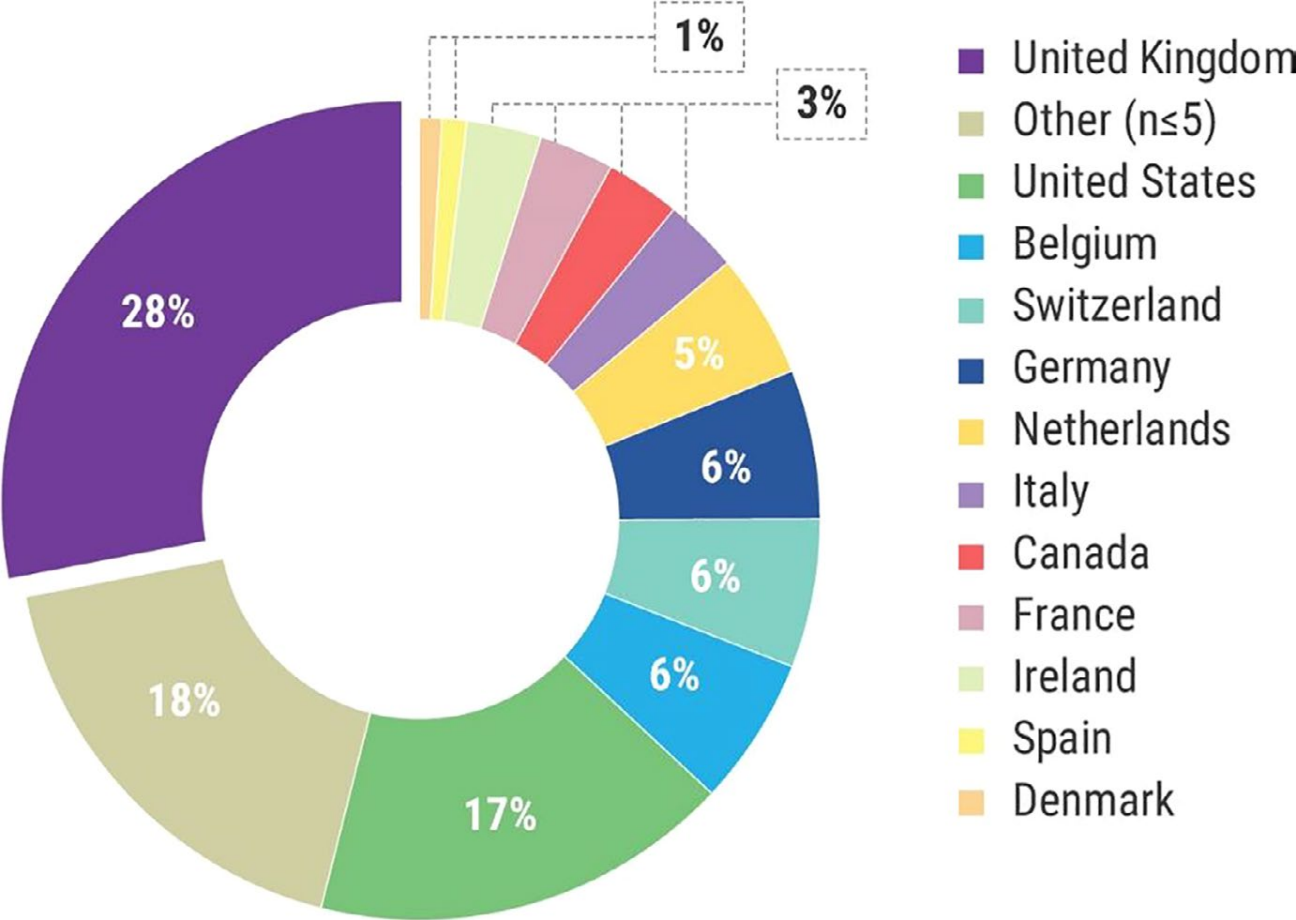
Total Survey respondents per country (Counts (N)). The group ‘Other’ comprised of countries with n=≤5 respondents each from; Afghanistan, Albania, Australia, Austria, China, Cyprus, Czech Republic, Finland, Greece, Hong Kong, India, Indonesia, Israel, Japan, Kazakhstan, Lithuania, Luxembourg, Macedonia, Malaysia, Malta, Moldova, Nepal, Norway, Philippines, Poland, Portugal, Romania, Serbia, Singapore, Slovenia, South Africa, South Korea, Sweden, Turkey, Uganda, Ukraine

A total of 372 respondents completed the survey in English (Table 2). The largest respondent (stakeholder) group was the patient community (patients (including carers), patient advocates and/or patient organizations) (35.8%), followed by industry (34.9%). Respondents completed the survey from 48 countries – a majority from the UK and United States (28.2% and 16.9%, respectively) (Figure 2). The group ‘Other’ comprised 36 countries, both within and outside the European Union (18%, n ≤ 5 respondents from each).

**Table 2:** Survey respondents by stakeholder from the total of 372 English respondents

Stakeholder	Total number of respondents	Per cent of total (%)
Patient community [Patients (including carers) † Patient advocates, Patient organizations‡]	133 (45†/88‡)	35.8%
Industry (Pharma, SMEs, Biotech)	130	34.9%
Research and Academia	38	10.2%
Health‐care professionals (HCP)	25	6.7%
Health Technology Assessment bodies (HTA)	12	3.2%
Regulator or Policymaker	10	2.7%
Research Funder	3	0.8%
Other§	21	5.6%

Note

The stakeholder group patient community is differentiated into: Patients (including carers)†, and patient advocates and patient organizations‡. Health‐care professionals (HCP) – clinical academics, Health Technology Assessment bodies (HTA), industry (pharmaceutical companies (Pharma), small and medium enterprises (SME) and Biotechnology companies (Biotech)), other § (include, but not limited to individuals that identify as their primary affiliation of; charity, consultant, independent expert, think tank, industry association, social association, funder, NGO’s, or identified as having multiple relevant affiliations.

### 3.2 Survey responses

#### 3.2.1 What is the status quo of PE today?

**Current perception of PE is low but ideal expectations are high.**

**Figure 3:**
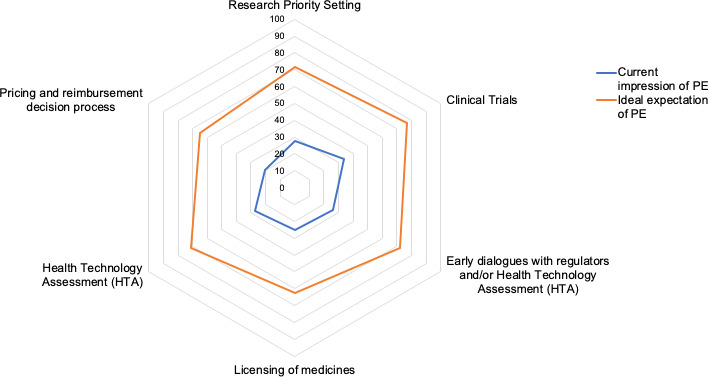
Current impressions vs ideal expectations of PE by key stage in the medicines development lifecycle. All stakeholders responded. Scale ranges from 0 (none) to 100 (ideal). Blue line is current impression, Orange line is ideal expectation over six stages of medicines development

Current expectations of PE were similarly low across all stages of the medicines’ lifecycle with the lowest expectations around HTA assessment, reimbursement and ED (Figure 3). Conversely ideal expectations of PE were similarly high across all six stages. The largest difference between current and ideal expectations of PE was at the ED stage.

#### 3.2.2 What are the challenges experienced with other stakeholders in their previous PE collaborations?

**Stakeholders acknowledge the challenge that patient input was not part of decision making.**

**Figure 4:**
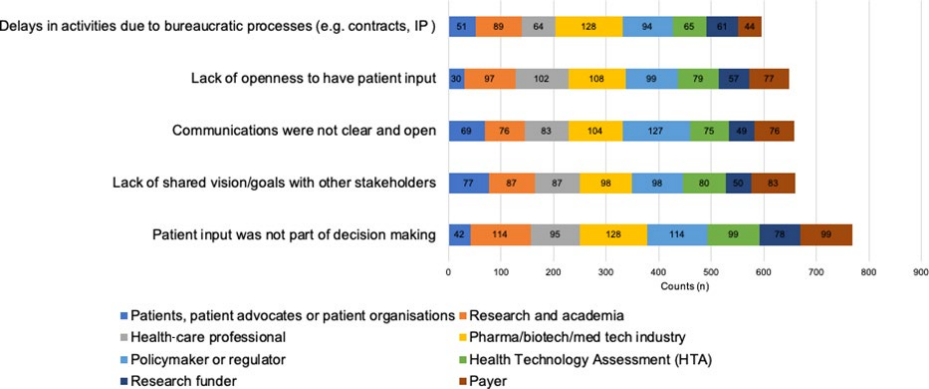
Previous challenges identified with other stakeholder groups with respects to PE activities (Total respondents). All stakeholders responded. Respondents could select more than one challenge per stakeholder group and could also report a challenge with their own stakeholder group

Of the 13 specified challenges in the survey question, the top overall challenge was, ‘Patient input was not part of decision making’. This was also the top challenge for research and academia, HTA bodies, research funders and payer groups. It was joint top with the industry group along with, ‘Delays in activities due to bureaucratic processes’. However, for the patient community this was less of an issue as the two top challenges were, ‘Communications were not clear and open’ and a ‘Lack of shared vision/goals’ (Figure 4).

### 3.3 What are the desired outcomes of PE?

**Greater patient‐centric input, patient‐relevant outcomes and communication are desired the most.**

Respondents were asked to indicate up to three most desired outcomes of PE from a separate list of predefined outcomes for each of the three stages. The top three are as follows (see also Supplementary Table 1 A‐C);

Research priority setting: i) When patients’ needs are leading in the research agenda, ii) when it results in new insights and new perspectives for policymakers and regulators and research funders and iii) when researchers get better insight in the patients’ journey.

Clinical trial design: i) When it results in more patient‐relevant outcomes for clinical trials, ii) when patients can share their experiences and increase knowledge of the clinicians and iii) when it leads to higher patient satisfaction during the trial.

Medicines licensing and Health Technology Assessment: i) When the voice of the patient is reflected in the decision, ii) when patients’ needs are better met and iii) when it results in improving transparency and openness in decisions.

#### 3.3.1 Are there dedicated organizational patient engagement functions within organizations and how are they utilized and supported?

Stakeholders feel prepared to engage in most PE activities, but require further support to do so.

**Figure 5:**
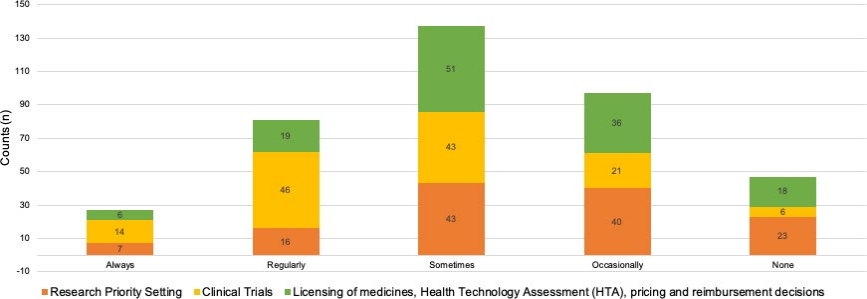
Proportion of industry dedicated to each of three stages of medicines development for patient engagement activities (Total counts). Industry respondents could select one of five options for each of the three key stages

A majority of industry respondents stated that they have a dedicated PE function in their organization (n = 99/130). The largest proportion of industry activities are ‘regularly engaged’ in clinical trial design (n = 46) and only ‘sometimes engaged’ at the research priority setting (n = 43) and at the licensing, HTA and pricing and reimbursement decisions stages (n = 51), respectively (Figure 5). Just over half of industry respondents stated they have a standard operating procedure (SOP) or other guidance on interactions with patients/patient organizations in place, and it is used in practice (n = 67/130). Conversely, three quarters do not have ‘Metrics or methods to determine impact of patient involvement’ (n = 103/130).

With respect to regulators, policymakers, HTA bodies and payers, and research funder groups, half of respondents (n = 12/23) reported that they have a dedicated PE function within their organization. Nearly, half of HCP respondents (n = 11/25) indicated that they do not have a SOP in place and it needs to be established and three quarters indicated that they do not have ‘Metrics or methods to determine the impact of PE’ (n = 20/25).

**Figure 6:**
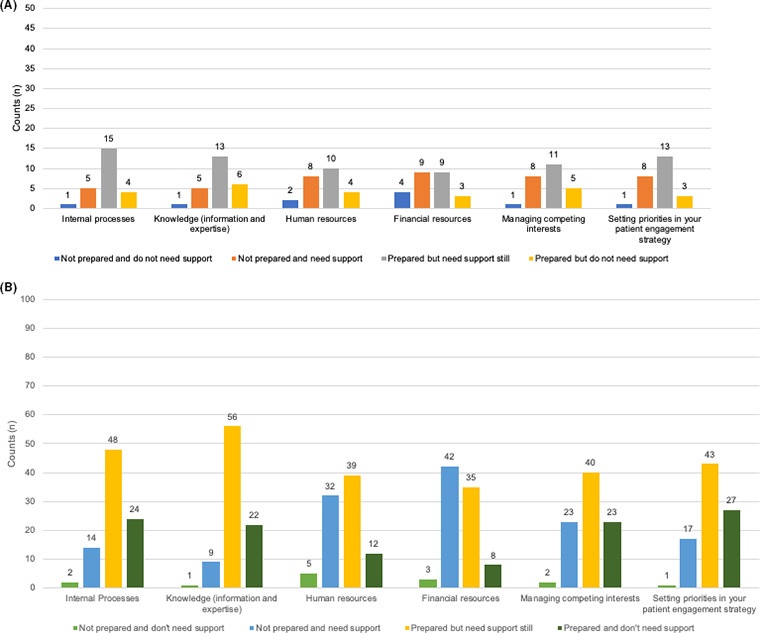
How prepared health‐care professionals (A) and patient organizations (B) are to undertake PE activities (total counts). HCP and PO respondents could select more than one process responses required at each of the four levels of preparedness in a matrix question

Additionally, patients and patient organizations, and HCP were asked if, ‘their organization was prepared to actively participate in PE’. Both stakeholder groups reported similarly that they are mostly ‘prepared but still require support’. Patients and patient organizations indicated that their greatest needs were ‘knowledge’ (n = 56) and ‘internal processes’ (n = 48). (Figure 6 A and B), yet HCP needed support similarly under every element listed (‘internal processes’, ‘knowledge’, ‘human resources’, ‘financial resources’, ‘managing competing interests’ and ‘setting priorities in your patient engagement strategy’).

#### 3.3.2 What is necessary to conduct more effective PE?

**All stakeholders need metrics to measure impact and better methods on how to do more effective PE.**

**Figure 7:**
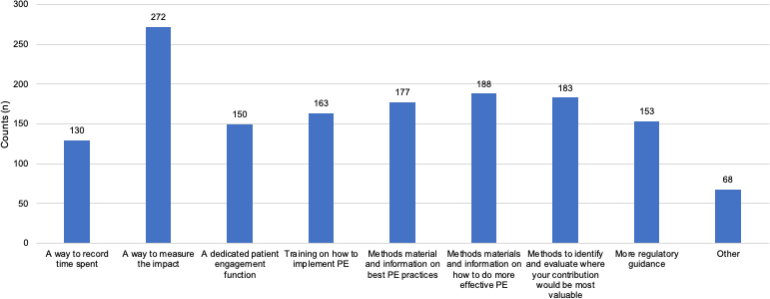
What is required to do more effective patient engagement (total respondents). All stakeholder responded. Respondents could select more than one option. In the ‘Other’ category, additional resources highlighted included ‘a clear framework and guidelines on how to engage with patients’ and ‘funding/ financial support from other stakeholders, particularly in providing funding/ reimbursement/tokens of gratitude for patients’

Out of the nine options given, *‘*A way to measure the impact’ was selected most frequently overall (n = 272) followed by, ‘Methods, materials and information on how to do more effective patient engagement*’* (n = 177) (Figure 7). When broken down by stakeholder group, stakeholder priorities differed slightly. The research and academia group most frequently reported that they required, ‘Training on how to implement PE processes in your organisation’ (n = 22), HTA respondents required ‘Methods, materials and information on how to do more effective patient engagement’ (n = 8), and the patient community, equally reported that they needed both ‘A way to measure the impact’ (n = 29), and ‘Methods to identify and evaluate where your contribution would be most valuable’ (n = 29).

#### 3.3.3 Is additional support for the patient community needed?

**Patients, patient advocates and patient organizations need additional dedicated support.**

Overall the patient community indicated that their greatest preference was for, ‘One‐to‐one support given directly to individual patients’ (n = 77/133). Specifically, a majority of patient respondents (ie not including patient organizations/advocates) indicated they would prefer support from a, ‘Person or group with in‐depth knowledge about the area of the planned engagement’ (n = 28/45), while a majority of patient advocates or patient organization respondents would prefer, ‘Other stakeholders who are responsible for the activity I (we) want to engage in’ to provide support (n = 61/88) (for full details see ^29^).

**Consultations:** *Increasing and supporting involvement of potentially vulnerable populations in PE*.

**Incorporating the lived experience and accommodating reasonable adjustments help to recognize and acknowledge the value patients bring.**

Several common barriers to increasing and sustaining patient involvement emerged from the consultations with young people, and people with dementia and their carers, along with recommendations to overcome these (Table 3).

**Table 3:** Major themes identified from two separate consultations (people with dementia (and their carers) and young people) and how to improve patient engagement (PE) with these populations. Other themes and full results are available at ^28^

Major themes raised (When involving patients)	Rationale for identified theme	Meaningful methods to enhance meaningful PE in patient populations according to the input provided
Voice of the person with the condition	*People with dementia* The people with the condition are the ‘experts by experience’ and their input is unique. *Young people* The people with the condition are the ‘experts by experience’ and their input is unique.	**Redress misconceptions** *People with dementia* Myths/misconceptions surrounding the type of disease, ability/willingness to participate, need for support. *Young people* Misconceptions about unable, or unwilling to contribute properly to PE. The early engagement in a research priority setting is feasible; the experience and/ or support young patients can provide in the design of clinical trials protocols, participation in regulatory activities is feasible if a suitable framework is established.
Diversity of patients involved and inclusion	*People with dementia* Account for differences such as age group, country, type and stage of dementia – mild, moderate and advanced dementia etc *Young people* Account for age group, country, different or complementary expertise to that of parents.	**Equal opportunities for patients to participate by ensuring accessibility needs are met** *People with dementia* The structure and format of the PE activity should consider the needs of the person with dementia: the accessibility of the materials received, the use of plain language and avoiding the use of jargon, acronyms and highly technical terms. Young people Include age appropriate formats and language.
Raising awareness of PE opportunities	*People with dementia* Provision of relevant information, support and training to patients, carers and other stakeholders interacting with them *Young people* Promoting autonomy, respect and equality. Ethical principles and the children's rights need to be considered in the design of involvement activities for children and young people.	**Reasonable adjustments for travel/accommodation, accessible information, training sessions/personal support, financial support/reimbursement** *People with dementia* Travel and accommodation costs incurred should be covered for both the person with dementia and his/her carer. Organizer of the PE activity should designate a ‘named person or a single point of contact’ with whom the person with dementia could speak to freely Accessible and understandable information about the PE activity *Young people* Travel and accommodation costs incurred should be covered for both the young person and their parents. Timing so that it doesn't interfere with school lessons Educational material and information (written and verbal) should be education level with appropriate language. A facilitator from a YPAG should be available to provide the right personal support to a young person in both the preparation and during the PE activity.

Understanding and incorporating the ‘lived experience’ of the patient (and not just the parent or carer) was considered to be paramount in adding genuine value to a given PE activity and in reflecting the expected outcomes. This can also serve to readdress many misconceptions of people with dementia and young people – namely that they are not able to or willing to be involved, an incorrect understanding of the condition(s) that they are living with, the added value that they can contribute, and the specific considerations (physical, mental, socioeconomic) of these populations.

The diversity of the patients involved in any PE activity was considered important to allow the broad range of experiences patients could contribute and provide equal opportunities for those who can be involved to do so. For example, different age groups, gender, cultural background and for people with dementia, the type and stage of dementia, all present a diverse set of experiences to consider. The person(s) to be involved should have enough information and understanding of the PE activity itself, so that they can make an informed decision about participation and that they can be effectively engaged throughout. For example, information should be provided both before and during the PE activity in clear, accessible formats that are language and age appropriate, using enhanced text, contrast, short sentences and bullet points whenever possible.

Raising awareness and enhancing PE opportunities for people could be facilitated through modifications to structured protocols for PE. These include reasonable adjustments for travel and accommodation; reimbursement of incurred cost that includes carers or additional help needed, utilizing time slots that are outside of school hours for young people, and providing support and accessible information to patients and carers and/or training, if appropriate, ahead of the PE activity. General information provided in advance can include topic areas to be discussed and any additional questions about the topic in question. For the other stakeholder(s) that are managing the PE activity, there could be a single trusted point of contact for the patients and their carers to have access to throughout the PE process.

**Workshop:** *Taking better account of HTA bodies considerations of PE in ED*.

**PE in ED with HTA bodies is less established and requires better rationalizing and supportive methods.**

Overall HTA bodies reported that PE adds value to ED and that more could be achieved. It was nuanced that ED with HTA bodies was not considered a decision‐making time for any party, though it is designed to help industry make decisions on their development programs. In practice, it more closely resembles consultation with the chance for bilateral feedback and input when patients are involved. In this context, PE in ED with HTA bodies is heterogeneous. Some HTA bodies already have mature practices for PE in ED, such as NICE and CADTH and through EUnetHTA, yet many others do not, or are as yet not fully convinced of the true benefit of PE in ED. Barriers that still need to be addressed included difficulties identifying and reaching out to patients with the appropriate profile and capacity to take part, the general lack of resources across HTA bodies to administer PE processes, and the lack of adaptable tools to create a consistent and workable framework for PE during ED (*Bertelsen N, et al ,2020, manuscript in submission to Health Expectations*). Tools that needed to be created to address this imbalance include: improving the patient recruitment processes, patient interview guidance, a minimum standards framework for involving patients, and more transparent rationale for PE in ED through accessible exemplar case studies on how this is or can be achieved (Table 4).

**Table 4:** Major challenges identified with patient engagement (PE) in early dialogues (ED) † from a focus group with Health Technology Assessment (HTA) bodies

Common challenges of patient engagement (PE) in ED	Types of tools, resources and guidance needed to improve PE in ED
Patients are unable to make objective inputs	Patient recruitment processes including developing criteria and guidance to help HTA bodies find, select and enrol patients into the early dialogue processes.
Difficulties finding patients (appropriate profile and capacity)	
Details are too complex for patients to engage with	Patient interview guidance including interview guides, standard questionnaires and guidance on adapting them to particular early dialogue topics.
Conflicts of interest between patient organizations and industry	Minimum standards framework including a framework of methods with guidance on their use, guidance for meeting chairs and patients and adaptation to meeting formats to accommodate specific patient needs.
Lack of internal resources (time, financial)	
Lack of clarity to engage patients at the Early Dialogues stage	Rationale for PE in Early Dialogues including metrics that show the impact of PE, case studies demonstrating how this can be achieved, definitions of early dialogues and better articulated rationale for PE.

Note

Participants were: (Instituto Aragonés De Ciencias De La Salud (IACS) Spain, The National Institute for Health and Care Excellence (NICE) UK, Haute Autorité de santé (HAS)/ European Network for Health Technology Assessment (EUnetHTA) France/EU, Norwegian Medicines agency (NOMA) Norway, Reggio Emilia Local Health Authority (RER) Italy, Tandvårds‐ & läkemedelsförmånsverket or The Dental and Pharmaceutical Benefits Agency(TLV) Sweden, Canadian Agency for Drugs and Technologies in Health (CADTH) Canada. NB † Early dialogue is not considered a decision‐making time for any party. In practice it more closely resembles consultation with the chance for feedback and input (two‐way communication). Adapted from *Bertelsen* N*, et al, 2020, under submission to Health Expectations*

**Delphi Method:** *Translating identified needs, expectations and preferences into agreed criteria for enhanced PE*.

The first two Delphi rounds resulted in many criteria being modified or dropped (see ^28^). Notably, at the category level, *Sustainability* was dropped after round two for ED. While experts recognized the importance of *Sustainability* of PE practices in ED, it was of a lesser current focus compared to other stages of medicines development. For the other two key stages, *Sustainability* was ranked equally low. Conversely, the category, *Aims and objectives* (and related category, *Key elements of practice design*, which included aims and objectives*)* were ranked similarly high across all three key stages. Criteria within this category centred on that the aims and objectives of PE practices should meet the expectations of patients and/or focus on patients’ needs and interests. They should also be agreed upon up front by all and be understandable to all involved participants (see Supplementary Tables 2, 3, 4).

At the criteria level overall, there were differences in the wording and definitions, across the three key stages (Table 5), the main difference, however, was in their respective weightings. The following general criteria were thus fairly consistent in their definition, across categories and the three key stages:

**Table 5:** Final list of major categories and respective weighting for effective patient engagement (PE), based on the consensus of three panel three round Delphi method.

Setting research priorities		Designing clinical trials		Early dialogues with regulators and HTA	
Criteria	Weight	Criteria	Weight	Criteria	Weight
Key Elements of Practice Design	19.5	Aim and objectives	14	Aim and objectives	17.9
Resources	16	Patient engagement impact	14	Target participants involved in patient engagement	15.3
Evaluation of the PE Practice in Setting Research Priority	12	Target patients involved	12	Involvement and participation	14.7
Capacity Building	12	Legal and ethical consideration	11	Code of conduct	11.3
Patient Engagement Impact	11.5	Involvement and participation	11	Capacity building	11.1
Involvement and Participation	11	Resources	10	Resources	10.9
Code of Conduct	10	Capacity building	10	Patient Engagement Impact	10
Sustainability	8	Evaluation of the PE practice in the Design of Clinical Trials	10	Evaluation	8.8
		Sustainability	8		
Total	100	Total	100	Total	100

Note

The final Delphi round aimed to i) reach consensus among the disagreement items not obtained after the first and second round; ii) Merging and rephrasing criteria and categories*;* iii) weighting categories; iv) weighting individual criteria. Starting from a list of approx. 50 questions for each decision point, after round 3 this was reduced to an agreed 20‐25 criteria separately for each key stage of medicines development, positioned under 8 or 9 major categories. All weightings across categories and criteria within each category, equate to 100. The full breakdown of round 1 and 2 results can be seen at^28^.

### 3.4 Involvement and participation

Patients’ participation should be properly planned, taking into account timing requirements, accessibility and vulnerability. More specifically, it should consider specific patient's circumstances and characteristics linked to but not limited to possible physical or mental impairments, cultural background, age and other relevant features (eg recordings, virtual communication and use of language, format of meetings, the venue and information provided). Additionally, an up‐to‐date single point of contact or a named person with whom patients can communicate when needed for information and/or support, is made available throughout their involvement in the activity (eg during ED).

### 3.5 Legal and ethical considerations that govern PE – including a code of conduct

It is necessary to have any relevant policy directives, legal, ethics, governance requirements and/or regulatory framework about how to engage patients included as part of PE practices. More specifically, ensuring that codes of conduct are adhered to and conflicts of interest are addressed and managed through accountability and transparency. Notably, participants raised as yet unresolved considerations of how to effectively balance conflicts of interests with suitable patient participation.

### 3.6 Building capacity to support the PE process

The competencies that are required to perform the PE activity itself need to be identified and actioned appropriately (eg in setting research priorities by all participants). Any training material should be adapted, comprehensible and accessible to all involved participants and take into consideration any impairments, literacy levels, cultural backgrounds and the circumstances of potentially vulnerable patients involved in setting research priorities.

### 3.7 Evaluation of the PE process

Generally, methods, tools and monitoring systems should be in place to evaluate the PE practice, including a framework. An evaluation of the outcomes should be linked to the aims and objectives of the PE practice in each setting. Additionally, the outcomes should be shared with all the participants involved and procedures should be in place so that the conclusions of the evaluation are used to support a continuous improvement process.

### 3.8 Synthesis of findings

#### 3.8.1 Stakeholders’ needs, expectations and preferences

Stakeholders continue to acknowledge that PE is crucial throughout the medicines development process and it needs to improve.^2^, ^3^, ^8^ There is a strong desire that PE has a greater focus on incorporating patient insights and expertise, that it results in better patient‐relevant outcomes, and that the patient voice is reflected in the decision‐making process. This last point was the top rated difficulty identified, that is, that patients are not involved in a decision‐making process; they are more often engaged at a lower level of involvement.^30^ This is nuanced in the case of ED with HTA, in that unlike other HTA processes where patients are involved, ED is not in itself a decision‐making process, so the expectations for HTA bodies and patients here need to be considered differently while sharing best practices globally. Frameworks to measure impact, a return on engagement and methods to undertake more effective PE, that are supported by exemplars of good practice, were needed most. Yet the patient community's needs focused more on the need for better communication and an alignment of shared vision. This may reflect the current focus and greater experience at CTD compared to other key stages of RPS and ED. It could be argued that the focus at CTD is partly driven by the desire to demonstrate quantitative downstream effectors [of PE] such as time and budget savings and increased recruitment rates,^19^ whereas at RPS and ED stages more useful measures are qualitative and more reflective of patient‐centred outcomes (such as empowerment and equality) and patient‐relevant research or endpoints.^31^, ^32^ Improvements at the RPS and ED stages could be best focused on more upstream effectors on methods and structures on how to do PE more effectively and that better align with patients’ expectations. At a strategic level, alignment between stakeholders on the positive rationale for involving patients in RPS and ED and how to effectively incorporate or utilize patient insights in their work can continue through EU and global platforms.^33^, ^34^, ^35^, ^36^, ^37^

Understanding and incorporating the lived experience of the patient was considered a paramount expectation for effective engagement. For the populations included here – people with dementia and young people, systematic efforts are limited by many of the reasons reported here.^17^, ^38^ Combining the results here with recent advances involving minors in medicine development within The publication ‘Principles on the involvement of young patients/consumers within the European Medicines Agency (EMA) activities’ ^11^ has established the first framework at the European level to promote the involvement of minors in medicines development from the regulatory perspective and can be built upon by others. and existing training platforms like the European Patients Academy (EUPATI) or the European Organisation for Rare Diseases (EURORDIS) Open Academy^39^ provide training of patients that can be supplemented by additional specific methodologies for interacting with potentially vulnerable populations, for example through KIDS Barcelona.^40^

### 3.9 Stakeholder support, resources and training

Most stakeholders felt confident and ready to engage in a PE activity, but need continued and improved support mechanisms to do so, especially on the ‘how to do better PE’. The patient community needs extra support through expert knowledge of the particular stakeholder and decision‐making process in question, and one‐to‐one mentorship of individual patients. This is currently often done through stakeholder specific networks and training programmes.^39^, ^41^ However, continued engagement with other stakeholder groups (such as regulators^11^, ^42^, ^43^, ^44^ HTA,^45^, ^46^ industry^47^, ^48^ working party and advisory groups^12^ ) can provide the much needed continuous bilateral knowledge exchange underpinned by a growing literature on successful multi‐stakeholder dialogues and engagement practices.^49^, ^50^ The variety of approaches by HTA bodies reflects a very different expectation, capacity and support needs by this stakeholder group to engage patients in ED. Patients are being involved in dialogues with some HTA bodies more consistently where resources allow; however, perceptual and practical barriers still exist across many HTA bodies. Leadership is gathering momentum through, for example, EUnetHTA and bodies like HTAi PCIG who share good practice and run workshops about PE in HTA at annual meetings. The rate of evolution here will likely be at a different rate from industry and regulators.

### 3.10 Enabling criteria for effective PE

The criteria identified here represent a common language and alignment across stakeholders on engagement approaches across the life cycle of medicines development. Despite some obvious and understandable stakeholder divergence on the categories and criteria for optimal PE, there was convergence of some of the weighted categories and criteria within them across the three stages of medicines development. Namely, the strong agreement upfront in the design part of the PE practice (ie aim and objectives) suggests that good PE can emerge as a consequence of good practice design. The full considerations of all patients involved be accounted for in the planning, implementation, reflection, evaluation and communication phases of the PE process. This includes improving inclusivity of, and accounting for, specific needs of potentially vulnerable populations. The PE approach itself is underpinned by adherence to all relevant ethical and legal considerations that are relevant for, available to and understandable by patients in a clear and accessible way. Measuring the value of the engagement itself – the return on engagement – should be substantive, logical and prospectively planned. Importantly, all Delphi criteria were agreed as important; thus, all should be taken into account during the planning, implementation and reflection of PE activities as part of the required cultural and practical evolution of addressing many of the barriers to PE.^51^

### 3.11 Co‐creation and coordinated actions for the evolution of PE

Creating methods, frameworks and guidance that address the varying stakeholder needs are complex. ‘One size does not (and should not) fit all’, and no single leader is needed. Instead, collective leadership and the co‐creation of solutions are needed via a ‘building block’ approach. These foundations are built on a detailed understanding of needs, expectations and preferences for effective PE in a multi‐stakeholder environment – as described here. This is complemented by demonstrating the value of PE more definitively through a monitoring and evaluation framework that is relevant, context specific and impact can be demonstrated through real case studies combined with materials adaptable to stakeholder needs.^52^ Combining these with other practical elements such as tools, and clear signposting to stakeholder specific material within regional and national networks^33^, ^34^, ^53^, ^54^, ^55^, ^56^ will enable an approach to PE that is most appropriate to the stakeholder(s) involved and specific question(s) being asked.

From the reported perspective of HTA bodies, the proposed ‘building block’ approach allows work to progress in parallel and at a different rate to other stakeholders and geographies, but remain linked and sympathetic to an evolving ecosystem approach to PE.

### 3.12 Limitations

The online survey was completed only by those who are familiar with and access to the technology, and a good understanding of English. The survey included questions that covered PE generally across medicines development and at three specific stages; these included broad and specific HTA process involving patients. Although piloted, it was impossible to identify how respondents interpreted the questions in the wider context, and where English was not their first language. Despite snowball techniques being used to maximize stakeholder reach, responses were dominated by patient and industry communities, and the central EU and North America regions; thus, the interpretation of conclusions for other stakeholder groups and countries outside these regions should be taken cautiously. Extending such a survey to non‐English languages would enrich applicability of future research findings. A temporary technical issue with the survey administration meant that we purposefully excluded any partially completed responses from the analysis. This resulted in reduced total response numbers, although we exceeded the minimum required response number of 370.

The consultations were relatively diverse with respect to experience of medicines development, geography, and for those with dementia, a range of dementia types. However, hard to reach groups and ethnic minorities may not have been sufficiently covered. Health‐care professionals represent a diverse group (general practitioners, nurses, clinical investigators/academics and pharmacologists). However, taking into account the low level of maturity towards PE, their diversity and the 3 decision points chosen here, we prioritized clinical academics. There are a vast number of HTA bodies across the EU and North America, some currently active in ED, many not. A generalization of reported themes across all HTA bodies and geographies is not appropriate at this time.

Finally, there were dropouts of Delphi panellists during the process for a variety of reasons. In the CDT Delphi group, many respondents were from North America; hence, some bias in interpretation of responses and weightings was inevitable. Despite this, the overall number and expertise balance within and between each group was monitored, and stakeholders' representativeness were not at risk.

## 4 CONCLUSION

This multi‐stage study adds significant value by building on some of the previously identified gaps between stakeholder specific understanding and importance of PE, and who should be responsible for leading efforts to improve it.^8^, ^15^, ^31^ These findings provide detailed and tangible building blocks to all involved stakeholders as to where stakeholder needs and expectations are, and where greater alignment could occur. The work of the PARADIGM consortium and its partners continue to address many of these signals holistically through multi‐stakeholder mechanisms^35^, ^37^, ^57^ in the continued evolution of meaningful, ethical and sustainable PE.

## CONFLICT OF INTEREST

The following authors have no conflicts of interest to disclose:

SDF, SSi, CP, FS, MJVE, LPR, ADP, DG, EF, BF, NB, AM, MB and NF.

NB works with a variety of stakeholder groups on patient involvement in health‐care decisions. This includes work as a consultant to patient groups and to the industry.

vii) This work was funded under EU IMI grant agreement number 777 450 – PARADIGM (Patients Active in Research and Dialogues for an Improved Generation of Medicines).

## 5 AUTHOR CONTRIBUTIONS’

All authors contributed to the design of this study and the writing of this paper.

Authors SDF, SSi, CP, MJVE, LPR, ADP, DG, EF, BF and NB also contributed to data analysis.

## Supporting information

Table S1‐S4

## Data Availability

The data that support the findings of this study are available from the corresponding author upon reasonable request.
