# Supplementary material for: Understanding multi‐stakeholder needs, preferences and expectations to define effective practices and processes of patient engagement in medicine development: A mixed‐methods study
Source: Health Expect. 2021 Feb 17;24(2):601–16. doi: 10.1111/hex.13207 (PMC8077078; doi:10.1111/hex.13207)
Supplement: Supplementary file 1 — Table S1‐S4 [file HEX-24-601-s001.docx]

**SUPPLEMENTARY MATERIAL**

| **Reported desired outcome of PE in research priority setting** | **Total number of responses (N)** |
| --- | --- |
| When patients' needs are leading in the research agenda | 218 |
| When all parties come to agreement early in the process | 110 |
| When patients also have a say in what research gets funded | 113 |
| When it results in new insights and new perspectives for policy makers and regulators and research funders | 214 |
| When researchers get better insight in the patients' journey | 188 |
| When it results in mutual learning | 121 |
| When patients receive feedback about the impact of their engagement | 105 |
| Other | 17 |

| **Reported desired outcome of PE in clinical trial design** | **Total number of responses (N)** |
| --- | --- |
| When information is better communicated to patients | 96 |
| When patients can share their experiences and increase knowledge of the clinician | 108 |
| When it results in more patient-relevant outcomes for the clinical trial | 267 |
| When it improves recruitment | 84 |
| When it improves diversity in recruitment | 33 |
| When it leads to reduced drop-out rate | 49 |
| When it leads to better compliance | 53 |
| When it leads to fewer protocol amendments | 34 |
| When it leads to shorter timelines of trials | 95 |
| When it leads to an earlier stop of unsuccessful research | 61 |
| When it leads to higher patient satisfaction during the trial | 106 |
| When patients receive feedback about the impact of their engagement | 87 |
| Other | 13 |

| **Reported desired outcome of PE in medicine licensing and health technology assessment (HTA)** | **Total number of responses (N)** |
| --- | --- |
| When all parties come to agreement early in the process | 103 |
| When the voice of the patient is reflected in the decision | 265 |
| When patients' needs are better met | 245 |
| When it leads to reduced decision time | 103 |
| When it leads to less reverted decisions | 24 |
| When it leads to better adherence (after market introduction) | 92 |
| When it results in improving transparency and openness in decisions | 181 |
| When patients receive feedback about the impact of their engagement | 57 |
| Other | 13 |

**Supplementary Table 1 Desired outcomes for PE at each of research priority setting (A), clinical trial design (B) and medicine licensing and health technology assessment (HTA) (C) (Total number of responses)**

Respondents selected from a predefined list for each of the three stages their top desired outcomes for PE. Respondents could select more than one option for each option.

| **Category**  **Name** | **Category weight** | **Criteria** | **Criteria weight** |
| --- | --- | --- | --- |
| Key elements of Practice design | 19.5 | The aim and objectives of the practice in setting research priorities are agreed and understandable by all stakeholders (e.g. patients, academics, researchers, pharmaceutical companies etc) involved and related to patient's needs and interest. | 26 |
|  |  | The practice is based on an assessment of the patient's needs and preferences. | 22.5 |
|  |  | Any relevant policy directives, legal, ethics, governance requirement and/or regulatory framework about how to engage patients in setting RP have been considered. | 11.5 |
|  |  | The patients engaged in setting research priorities reflect diversity of the target population †, patients' circumstances‡ and vulnerability. | 14.5 |
|  |  | The roles and responsibilities are clearly defined, agreed and assigned among all participants. | 12 |
|  |  | The practice includes specific tools§, and mechanisms¶, to ensure that all participants understand their own and other’s roles and responsibilities. | 13.5 |
| Code of conduct | 11.5 | The practice includes a code of conduct, which clearly states the rules and procedures, including ethical principles, of participation in setting priorities for all stakeholders. | 44 |
|  |  | The terms and conditions of confidentiality agreements are in place, presented in a clear and accessible way to the target population involved. | 30 |
|  |  | The practice contains clear and accessible mechanisms to manage potential conflicts of interest. | 26 |
| Resources | 10 | All participants are informed of the available resources, including equitable financial compensation framework to support them during the process (e.g. travel and subsistence expenses, time missed from work, child/elderly care etc.). | 27.5 |
|  |  | The infrastructures needed for participants' involvement are in place^1^ and adapted to specific circumstances of participants^2^ | 41 |
|  |  | The practice includes guidance, ready-to-use tools and templates material to facilitate effective patient engagement implementation. | 31.5 |
| Capacity building | 11 | The practice identifies the competencies that are required to perform patient engagement in setting research priority by all participants. | 34 |
|  |  | The training material is adapted, comprehensible and accessible to all participants taking into consideration impairments, literacy levels, cultural background and the circumstances of vulnerable patients involved in setting research priority. | 35.5 |
|  |  | The practice ensures that all stakeholders (other than patients) are adequately trained for their role. | 30.5 |

| **Category**  **Name** | **Category weight** | **Criteria** | **Criteria weight** |
| --- | --- | --- | --- |
| Involvement and  participation | 12 | Patients receive timely, clear, accessible and appropriate information about their involvement and what is expected of them. | 26 |
|  |  | The patient's participation will be properly planned, taking into account timing requirements, accessibility, and vulnerability. | 18.5 |
|  |  | Whenever appropriate, and as agreed by participants, regular feedback is communicated in a clear and adapted way. | 20 |
|  |  | All participants are given the opportunity to give regular feedback about the process. | 19 |
|  |  | The practice ensures there is a named key contact that patients can reach out throughout the process. | 16.5 |
| Evaluation of the PE practice in setting research priority | 12 | The evaluation framework is included and shared among the participants. | 15.5 |
|  |  | Methods, tools and monitoring system are in place to evaluate the PE practice. | 22 |
|  |  | The evaluation outcomes are linked to the aim and objectives of the PE practice in setting research priority. | 24.5 |
|  |  | The evaluation outcomes are shared with all the participants and feedback is enabled after the completion of the process. | 14.5 |
|  |  | The practice identifies SMART ^3^ relevant outcomes regarding the involvement of patients in setting research priority | 23.5 |
| Patient engagement impact | 16 | Research topic and priorities become more appropriate, based on patients’ needs. | 22 |
|  |  | The product profile (drug, technology) become more relevant and usable for patients. | 19 |
|  |  | Research questions and outcomes/endpoints become more relevant for patients. | 19.5 |
|  |  | Credibility of submitted research proposals are enhanced when involving patients and patient organisations. | 10.5 |
|  |  | Resource allocations when engaging patients within industry become more appropriate and are based on patients’ needs. | 12.5 |
|  |  | Completed studies will provide more useful information for patients and decision-making process when involving patients and patient organisations. | 16.5 |
| Sustainability | 8 | The practice is embedded in the institution or organisation and when relevant, the alliances with private and/other public institution are fostered. | 46 |
|  |  | Human and financial resources are allocated for the long-term continuity of the practice on PE in setting research priorities. | 31 |
|  |  | There is a dissemination and communication plan demonstrating the process and outcome of PE. | 23 |

**Supplementary Table 2 Final list of major categories, criteria, and their respective weighting for effective patient engagement (PE), at research priority setting (RPS).**

Final criteria and categories were agreed by the expert panel after 3 rounds of Delphi. All weightings across criteria and categories within each criteria, equate to 100.

† *Refers to cultural background, social-economic status, gender, age, ethnicity, educational level, disease, disability and vulnerability.*

‡ *Former patients, patients who are at risk for a disease but do not yet have the disease, severity of the disease, patient’s advocate, carers, patient’s representatives, patient organizations, level of experiences, patient's scientific knowledge/background).*

§ *(e.g., roles and responsibility charts, procedural guidance documents, protocols)*¶ *(e.g., meetings, workshops, training sessions)*

*1(e.g., technological support, working space, communication technologies)
2(e.g possible physical, mental, cognitive or any impairment, etc).*

^3^*SMART: Specific Measurable Achievable Realistic Time bound*

| **Category**  **Name** | **Category weight** | **Criteria** | **Criteria weight** |
| --- | --- | --- | --- |
| Aims and objectives | 14 | There is general agreement on the aims and objectives of the practice on PE and these are understandable by all relevant stakeholders involved in the design of CT. | 40.6 |
|  |  | The aims and objectives of the practice on PE focus on patient’s needs and interests. | 34.7 |
|  |  | The timeline for the engagement in the design of clinical trials is appropriately planned and allows for incorporation throughout the process. | 24.7 |
| Target participants involved | 12 | The patient representatives†, engaged in the design of clinical trials reflect the relevant diversity ‡, of the target population. | 32.9 |
|  |  | The practice includes a clear description of the process and criteria followed to identify patient representatives to participate in the design of clinical trials. | 19.0 |
|  |  | The role and responsibilities of all target participants involved in the design of clinical trials, including patient representatives, are clearly defined and agreed. | 23.7 |
|  |  | The practice includes a clear description of the process followed§ to make sure that all participants understand their own and other's roles and responsibilities. | 24.4 |
| Legal and ethical consideration | 11 | The practice includes a code of conduct, which clearly states the principles of participation in design of clinical trials for all stakeholders involved. | 18.9 |
|  |  | The practice contains procedures to identify and address unethical behaviours, towards all stakeholders, during their participation in the design of the clinical trials process. | 10.9 |
|  |  | There is a clear description of efforts made to ensure that all stakeholders involved know the principles of participation in clinical trials design (e.g., workshops, meetings). | 11.0 |
|  |  | The terms and conditions of all legal agreements are written and communicated in a clear and accessible way and adapted to the target population involved. | 14.2 |
|  |  | Potential conflicts of interest are addressed and managed (up to avoidance). For this, policies that require full disclosure, transparency and accountability are developed. | 15.7 |
|  |  | Funding resources for the practice on PE in the design of clinical trials are clearly documented and explained to all stakeholders involved prior to involvement, and any changes that occur during the practice are communicated up-front. | 14.0 |
|  |  | All relevant policy directives, legal and/or regulatory framework have been followed when engaging patients in the design of clinical trials. | 15.3 |

| **Category**  **Name** | **Category weight** | **Criteria** | **Criteria weight** |
| --- | --- | --- | --- |
| Resources | 10 | All participants involved in the design of clinical trials practice are informed, in clear and accessible way, of the resources available to support their tasks during the process. | 19.3 |
|  |  | A clear, transparent and equitable financial compensation framework is in place for patient representatives who participate in the PE practice in the design of clinical trials (e.g., reimbursement of expenses for travel, time missed from work, subsistence, child/elderly care, stipends). | 24.1 |
|  |  | Funding is allocated to cover governance, administration and operations of the practice in PE on design of clinical trials. | 18.6 |
|  |  | Infrastructure is in place to support the practice in PE on design of clinical trials and is adapted to specific circumstances of patient representatives (e.g. technological support, working space, information and communication technologies). | 21.4 |
|  |  | The resources include guidelines, and ready-to-use tools and templates, material that facilitates effective patient engagement implementation in a clear and accessible way. | 16.6 |
| Capacity Building | 10 | Comprehensive and accessible trainings or induction materials and programs are available to respond to the needs of the patient representatives involved in the design of clinical trials. | 54.8 |
|  |  | Practices on PE in design of clinical trials incorporate training/coaching for stakeholders, other than patient representatives, regarding how to incorporate patients in the design of clinical trials. | 45.2 |
| Involvement and Participation | 11 | The type of interaction among the different stakeholders is defined at each stage (e.g., co-creation, advice, consultation). | 18.4 |
|  |  | There are mechanisms Including documents, charts, guidance, training, meetings, and workshops ¶ in place to ensure participation of patient representatives, taking into consideration participant’s characteristics, and circumstances of vulnerable population. | 29.4 |
|  |  | Patient representatives receive timely and regular feedback about the outcomes/changes for each phase of the involvement, in a clear and understandable format and adapted to patients' circumstances. | 31.7 |
|  |  | A clear point of contact is available to whom patient representatives can reach out to when needed for information and/or support, throughout their involvement in the design of clinical trials. | 20.6 |
| Evaluation of the PE practice in the design of clinical trials | 10 | There are methods, tools and monitoring systems in place to evaluate the PE practice. | 24.2 |
|  |  | The evaluation criteria are linked to the aims and objectives of the PE practice. | 23.3 |
|  |  | The evaluation outcomes are shared with all the stakeholders involved in the practice using appropriate channels and formats suited to patient perspectives' circumstances and needs. | 25.6 |
|  |  | The evaluation outcomes are used to improve future PE practices for design of clinical trials. | 26.9 |

| **Category**  **Name** | **Category weight** | **Criteria** | **Criteria weight** |
| --- | --- | --- | --- |
| Patient engagement impact | 14 | Improved recruitment. | 7.2 |
|  |  | Improved retention (i.e fewer drop outs) of study participants. | 6.8 |
|  |  | Better adherence to the research protocol. | 7.1 |
|  |  | Fewer amendments to the research protocol. | 7.3 |
|  |  | Improved trial experience for study participant. | 11.1 |
|  |  | More timely study completion. | 7.3 |
|  |  | More inclusive, sensitive and ethical trial design, which are appropriate for needs and circumstances of the target population. | 12.4 |
|  |  | The wording and timing of research instruments (e.g. questionnaires and interventions) are appropriate for specific needs and circumstances of the target population. | 9.8 |
|  |  | Patient information and education materials (e.g. lay summaries, information and education sheets, consent form, recruitment material) are appropriate to the specific needs and circumstances of the target population. | 10.9 |
|  |  | Additional study-specific relevant measures are included to complement the described ones. | 5.6 |
|  |  | Identification of meaningful endpoints for patient representatives. | 14.5 |
| Sustainability | 8 | The continuation of the practice is ensured through ownership and it is systematically embedded in the institution or for all stakeholders. | 47.8 |
|  |  | Human and financial resources required for the long-term continuity of the practice on PE in the design of clinical trials are identified and secured. | 52.2 |

**Supplementary Table 3 Final list of major categories, criteria and their respective weighting for effective patient engagement (PE), for Clinical Trial Design (CTD).**

Final criteria and categories were agreed by the expert panel after 3 rounds of Delphi. All weightings across criteria and categories within each criteria, equate to 100.

†Patients, carers, parents or tutors and/or staff from patient’s organisations that act as a liaison between the patients and other organisations, represent their interest and advocates for the rights.
‡Including but not limited to cultural background, socio-economic status, gender, age, ethnicity, educational level, disease, disability, vulnerability, etc.

§ Including documents, charts, guidance, training, meetings, and workshops.

¶ (e.g., language, format of meetings, the venue, time of the day, etc.)

| **Category**  **Name** | **Category weight** | **Criteria** | **Criteria weight** |
| --- | --- | --- | --- |
| Aim and objectives | 17.9 | The practice aims to meet patients' expectations when participating in the early dialogue process. | 46.5 |
|  |  | The aim and objectives of PE in Early Dialogues are clear and understandable for all stakeholders involved. | 53.5 |
| Target participants involved in patient engagement | 15.3 | The practice of PE in early dialogues includes rigorous methods to identify patients or patient representatives † from the relevant target population needed for a particular dialogue. | 30.5 |
|  |  | The process captures the diversity of the target population and their range of perspectives. | 25 |
|  |  | Relevant points of view other than those patients (e.g. carers, parents etc.) are also considered. | 16.5 |
|  |  | Guidance and adequate training are provided to ensure that the role and responsibilities of all stakeholders involved are clearly defined and understood by all. | 28 |
| Code of conduct | 11.3 | Any applicable policies, legislative and regulatory frameworks (e.g. EMA) about when and how to engage patients in early dialogues with HTA and regulators have been followed | 26.5 |
|  |  | The practice includes a code of conduct, which clearly states the rules of participation in early dialogues for all stakeholders involved. | 25 |
|  |  | The terms and conditions of all policies and confidentiality agreements are adequate, clear and accessible. | 16.5 |
|  |  | The PE practice incorporates mechanisms to disclose and manage all potential conflicts of interest of all stakeholders. | 18 |
|  |  | The PE practice contains procedures to identify and address potential discriminatory, coercive, intimidating, and unethical behaviours before, during and after the participation. | 14 |
| Resources | 10.9 | The practice uses relevant PE methodologies, guidance and tools. | 20.2 |
|  |  | The practice includes a fair financial framework‡ for participating patient representatives. | 19.7 |
|  |  | Sufficient funding is in place to ensure that all the elements of the practice are covered in its’ entirely. | 19.7 |
|  |  | Financial arrangements for the practice of PE in early dialogues are clearly explained to all stakeholders involved, prior to involvement, and any changes that occur during the practice are communicated up-front. | 17.2 |
|  |  | An adequate infrastructure is in place (e.g., technological support, working space, information and communication technologies) and adapted to specific requirements of participants (e.g. possible physical mental, cognitive or any impairments etc.). | 23.2 |
| Capacity building | 11.1 | The practice on PE describes the relevant competencies, expertise and experience required by all stakeholders to effectively engage in this process. | 42.9 |
|  |  | The practice includes comprehensive and accessible training or induction material and programs to respond to the needs of the participants involved in early dialogues with HTA and regulators. | 57.1 |

| **Category**  **Name** | **Category weight** | **Criteria** | **Criteria weight** |
| --- | --- | --- | --- |
| Involvement and participation | 14.7 | The process respects the need for appropriate planning and preparation time, to allow patient representatives to effectively engage from the beginning. | 19 |
|  |  | The mechanism of interaction with patients’ representatives is adapted to their needs to ensure effective PE. More specifically, it considers specific patients’ circumstances and characteristics linked to but not limited to possible physical or mental impairments, cultural background, age and other relevant features (e.g., recordings, virtual communication, use of language, format of meetings, the venue, and information provided). | 28.5 |
|  |  | The practice on PE in early dialogues sets mechanisms to ensure a fair deliberative process* that allows equal opportunity for participants' contribution. | 16.2 |
|  |  | A regular feedback system is in place to inform patient representatives about the outcomes/changes, as appropriate, including the option for patients’ representatives to comment on the final output. | 10.8 |
|  |  | The information about the outcomes included for each phase of the involvement is communicated in clear and plain language, using accessible formats, and taking into account potential disabilities and impairments, as appropriate. | 10 |
|  |  | The practice includes an up-to-date single point of contact or a named person to whom patients can reach out to when needed, for information and/or support, throughout their involvement in early dialogues. | 15.5 |
| Evaluation | 8.8 | The practice includes an evaluation framework linked to the aim and objectives, including methods, tools and monitoring system, which enables systematic evaluation at appropriate phases of the process. | 29.8 |
|  |  | The practice identifies relevant outcomes regarding the involvement of patients in early dialogues with HTA and regulators. | 31.8 |
|  |  | The evaluation outcomes are shared with all the participants involved. | 16.6 |
|  |  | The practice includes procedures by which the conclusions of the evaluation are used to support a continuous improvement process. | 21.8 |
| Patient Engagement Impact | 10 | The practice identifies potential impact from the practice, for example:   - Better understanding of the impact of health technologies and treatments in real life context and the quality of life aspects. - More practical and better designed development plan for health technologies and treatments. - Better reflection of the needs and preferences of patients in development plans for new technologies. - Inclusion of patients’ relevant outcomes and end-points in clinical trials data collection and analysis. - Better quality of the evidence assessed during the later regulatory and HTA assessment. - Higher relevance of the advice given to the local context of application.   Transparency of regulatory and HTA processes leads to better understanding and trust of the scientific procedures. | n/a |

**Supplementary Table 4 Final list of major categories, criteria and their respective weighting for effective patient engagement (PE), for Early dialogues with regulators and HTA (ED).**

Final criteria and categories were agreed by the expert panel after 3 rounds of Delphi. All weightings across categories and criteria within each category, equate to 100. For category 8 Patient engagement Impact, the panel discussed the difficulties in measuring impact in this setting, thus provided a list of options that had no weighting assigned to them

*† Patients, vulnerable population, carers, parents or tutors and/or staff from patient’s organization that act as a liaison between the patients and other organizations, represent their interests and advocates for their rights.*

*‡ Mechanism for payment/reimbursement*
